# OX40 (CD134) Expression on T Regulatory Cells Is Related to Serious Hypertensive Disorders in Pregnancy

**DOI:** 10.3390/jcdd10100431

**Published:** 2023-10-17

**Authors:** Maciej Kwiatek, Agnieszka Kojak, Anna Kwaśniewska

**Affiliations:** Department of Obstetrics and Pregnancy Pathology, Medical University of Lublin, 20-059 Lublin, Poland; agnieszka.kojak@umlub.pl (A.K.); anna.kwasniewska@umlub.pl (A.K.)

**Keywords:** gestational hypertension, preeclampsia, T regulatory cells, OX40

## Abstract

Hypertension is one of the leading causes of morbidity and mortality among women related to pregnancy, childbirth and the postpartum period. The pathogenesis of gestational hypertension is complex and still not fully understood. The aim of this study was to assess the population of circulating CD4+CD25+FoxP3+ cells and its differentiation in terms of OX40 expression in two forms of hypertension: isolated hypertension developing after the 20th week of pregnancy and pre-eclampsia. The study included a group of 60 patients with hypertension and 48 healthy controls. The analysis of the percentage of Tregs was performed by flow cytometry. There was no difference in the percentage of peripheral lymphocytes between the groups. In the group of women with preeclampsia compared to the group with gestational hypertension, significantly higher percentages of CD4+CD25+FoxP3+ cells (*p* = 0.03) and percentages of CD4+CD25+FoxP3+ cells expressing the OX40 antigen (*p* = 0.001) were observed. OX40 expression on Tregs seems to be related to more serious type of hypertensive disorders in pregnant women.

## 1. Introduction

Hypertension is one of the leading causes of morbidity and mortality among women related to pregnancy, childbirth and the postpartum period. It is observed in about 5–10% of pregnancies [1,2], Hypertension is the reason for about 70,000 maternal deaths every year in the world [3]. The occurrence of blood-pressure disorders during pregnancy may result in long-term consequences for the woman, as they are a known risk factor for the development of future cardiovascular diseases, kidney diseases [2,4,5], metabolic disorders and cerebrovascular diseases [1,6]. The disease in a pregnant woman also leads to serious complications in the fetus and newborn. Hypertension in pregnancy increases the risk of intrauterine hypoxia and death, and is often associated with intrauterine growth disorders, preterm delivery and all the consequences of prematurity and low birth weight [2,5,7,8,9]. The classification of hypertension in pregnancy distinguishes four main forms: chronic hypertension, gestational hypertension clinically manifested in the second half of pregnancy, pre-eclampsia and pre-eclampsia superimposed on chronic hypertension.

The pathogenesis of gestational hypertension is complex and still not fully understood. One of the leading theories involves the immunological background of the disease [10]. Mechanisms responsible for maternal immune tolerance include placental expression of polymorphic MHC molecules, production of anti-inflammatory and protolerogenic hormones, cytokines and modulatory molecules, and specific immune regulation at the decidual level. Decidual NK cells and antigen presenting cells, among others, play a key role in the proper targeting of the maternal immune response. They have the ability to initiate the differentiation of naive T cells towards various subsets, such as Th1, Th2, Th17 and Treg lymphocytes, and maintain a subtle balance of the immune system [11]. Regulatory T cells are characterized by the CD4+CD25+ phenotype and the additional expression of the FoxP3 transcription factor is an indicator of their activity [12,13]. Under physiological conditions, the population of regulatory T cells plays a key role in the development and maintenance of peripheral immune tolerance by controlling the circulating autoreactive T cells that have not been eliminated from the thymus [14,15]. In addition, they are able to suppress inflammatory processes caused by infectious agents, causing temporary or permanent impairment of the functions of host tissues and organs, thus guaranteeing their safety [16,17]. They play a unique role in the events related to pregnancy and reproduction, protecting the fetus during pregnancy against rejection by the mother’s immune system [18]. Soon after fertilization, Tregs suppress the inflammatory process that occurs during implantation [19]. The population of decidual Tregs increases during early pregnancy and constitutes 10 to 30% of all CD4+ cells until delivery, when a marked decrease in the number of these lymphocytes is observed [12,20,21]. Interestingly, the population of Treg cells circulating in peripheral blood changes very similarly [22,23]. Disturbances consisting in insufficient recruitment of Tregs or their improper functioning may lead to impaired implantation [24,25] and abnormal trophoblast invasion within the spiral arteries [26,27], which in turn may lead to miscarriage, the development of hypertension in pregnancy and impaired fetal growth [28]. It should be noted that the Treg population is very heterogeneous and its composition changes with the duration of pregnancy [29,30]. Many different antigens, either receptors or cofactors, can be found on the surface of regulatory T cells, and their functions are still being explored.

The tumor necrosis factor receptor OX40 (CD134, TNFRSF4) is a member of the TNF receptor superfamily that is transiently expressed on activated T cells. The expression of the OX40 is characteristic mainly for activated CD4 and CD8 T lymphocytes, but its presence has also been demonstrated on other immune cells: NK cells, NKT cells, neutrophils and eosinophils [31,32,33]. OX40 is activated by the cognate ligand OX40L (CD252/TNFSF4/gp34), which is expressed mainly on antigen presenting cells (APCs) such as B lymphocytes, mature and plasmacytoid DCs and macrophages, but also on other cell types including Langerhans cells, endothelial cells, mast cells and NK cells [33,34]. The ligand of OX40 (OX40L: TNFSF4, CD134L) is mainly expressed on mature antigen-presenting cells as well as on vascular endothelial cells. OX40 is a pro-inflammatory receptor, inhibits differentiation and directly impairs the suppressive function of Tregs [35]. Its exact role in pregnancy has not been clearly defined.

So far, it has been proven that in pre-eclampsia, a significant reduction in the population of regulatory T cells is observed both in animal models and in humans [27,36]. The aim of this study was to assess the population of circulating CD4+CD25+FoxP3+ cells and its differentiation in terms of CD134 expression in two forms of hypertension: isolated hypertension developing after the 20th week of pregnancy and pre-eclampsia.

## 2. Material and Methods

The study included a group of 108 pregnant women. A total of 60 women were patients hospitalized in the Department of Obstetrics and Pathology of Pregnancy, Medical University of Lublin, due to pregnancy complicated by pregnancy-induced hypertension, including pre-eclampsia. The control group consisted of 48 women with normal, uncomplicated pregnancy.

### 2.1. Inclusion Criteria

The study group consisted of pregnant women diagnosed with gestational hypertension or pre-eclampsia. Gestational hypertension was diagnosed when systolic blood pressure and/or diastolic blood pressure was ≥140 mmHg, ≥90 mmHg occurring on two occasions at least 4 h apart after 20 weeks of gestation in a woman whose blood pressure has previously been normal. The diagnostic criteria for pre-eclampsia included hypertension in the second half of pregnancy in a woman with previously normal blood pressure and proteinuria (protein loss ≥ 300 mg in a 24 h urine specimen). In the absence of proteinuria, pre-eclampsia can be diagnosed when the new-onset hypertension is accompanied by the new onset of any of the following: thrombocytopenia, renal insufficiency, impaired liver function, pulmonary edema, neurologic disorders and/or fetal growth restriction. However, we have always observed proteinuria among our patients with preeclampsia (Table 1).

### 2.2. Exclusion Criteria

Women with chronic diseases, such as hypertension, diabetes, thyroid diseases, kidney diseases, allergic and autoimmune diseases and other clinically significant comorbidities, were excluded from the study. Patients with a diagnosis of threatening preterm delivery, premature rupture of membranes (PROM) and symptoms of infection were also excluded from this group. The study group did not include patients with signs and symptoms of pre-eclampsia superimposed on pregestational hypertension (Table 1).

#### 2.2.1. Isolation of Mononuclear Cells (PBMC) from Peripheral Blood

Peripheral venous blood was collected in a volume of 9 mL from the elbow bend into sterile S-Monovette tubes, in which sodium heparin was used as an anticoagulant (SARSTEDT, Nűmbrecht, Germany). The analysis of the percentage of Tregs was performed by flow cytometry (FACSCantoTM II, Becton Dickinson, San Jose, CA, USA) This cytometer is equipped with two lasers (argon laser—488 nm, diode laser—633 nm) enabling the simultaneous analysis of up to 8 fluorescent markers and the automatic Cell-Quest software v8.0.1. was used for cell phenotyping and evaluation.

The obtained tissue material was processed within 2–6 h of collection. The peripheral blood mononuclear cell (PBMC) population was isolated from whole blood by density gradient centrifugation. Peripheral blood was diluted 1:1 using 0.9% phosphate buffered saline (PBS; Biochrome AG, Berlin, Germany) under aseptic conditions in a laminar flow chamber with sterile air. The diluted blood was then layered on Gradisol L reagent (Aqua Medica, Poznan, Poland) and centrifuged for 20 min at 2800 rpm to separate the buffy coat of PBMC. Separated mononuclear cells settled at the interface were collected and transferred with a sterile Pasteur pipette to new tubes. After washing twice in PBS (centrifuged at 300× *g* for 5 min at room temperature), the supernatant was removed from the cell pellet. The final stage of the procedure was the assessment of the initial number of isolated PBMC cells by counting them in a Neubauer chamber. The medium used for cell freezing contained RPMI 1640 (Biochrome AG, Berlin, Germany) supplemented with 10% fetal bovine serum (FBS) (PAA, Paching, Austria) and 10% DMSO (dimethyl sulfoxide, Sigma Aldrich, Co., St. Louis, MO, USA). A 1.5 mL quantity of medium was added to the suspension of cells (approx. 10 million) and stored at −80 °C until analysis.

#### 2.2.2. Determination of The Immunophenotype of Regulator T Cells

Thawed mononuclear cells were washed twice in buffered saline by centrifugation for 5 min (RCF 300) at room temperature. After centrifugation, the supernatant was removed and the cells were resuspended in buffer of PBS.

The following set of monoclonal antibodies was used to assess the immunophenotype of Tregs subpopulations: Alexa Fluor^®^ 647 Mouse anti-Human FoxP3 (BD PharmingenTM, Franklin Lakes, NJ, USA), V450 Mouse Anti-Human CD4 (BD HorizonTM, Franklin Lakes, NJ, USA), PE-CyTM7 Mouse Anti-Human CD25 (BD PharmingenTM, Franklin Lakes, NJ, USA), FITC Mouse Anti-Human CD134 (BD PharmingenTM, Franklin Lakes, NJ, USA). The mixture was incubated for 20 min in the dark, then washed in PBS buffer without Ca^2+^ and Mg^2+^ ions by centrifugation for 5 min (RCF 300). The supernatant was then poured off and the cells were fixed in Cell Staining Buffer (BioLegend, San Diego, CA, USA). The fixed cells were resuspended in PBS, centrifuged for 5 min at 500× *g* and then the supernatant was removed.

In order to increase the permeability of cell membranes, the cells were incubated for 30 min at room temperature with a permeabilization buffer (FoxP3 Fix/Perm Buffer Set) (BioLegend, San Diego, CA, USA). After permeabilization, the cells were washed twice in PBS. Next, intracellular FoxP3 antigen was labeled using Alexa Fluor^®^ 647 anti-FOXP3 antibody at a concentration in accordance with the manufacturer’s procedure. After a 20 min incubation in the dark (RT), the cells were washed twice in PBS buffer. The supernatant was removed, and the resulting pellet of fluorochrome-labeled cells was suspended in 100 µL of PBS buffer and subjected to cytometric analysis. A total of 10^5^ lymphocytes were analyzed at a time.

**Figure 1:**
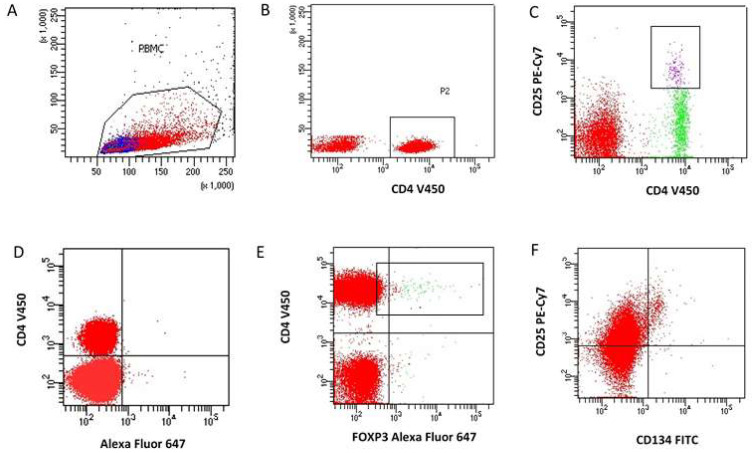
An example of cytometric analysis of Treg CD4+/CD25+/FOXP3+ lymphocytes with the expression of CD134 antigens among peripheral blood lymphocytes. (**A**). Dot plot in scattering coordinates (FSC/SSC). (**A**) linear scale is used on both axes; a lymphocyte separating region (PBMC) was created; (**B**). dot plot (dot plot), linear scale (SSC)/logarithmic scale (CD4 V450)—extraction of gate R2 containing CD4+ T cells; (**C**). dot plot (dot plot) logarithmic scale (CD4 V450/CD25 PE-Cy7) representing CD4+CD25+ regulatory T cells; (**D**). dot plot logarithmic scale (CD4 V450)/Mouse IgG1 Alexa Fluor 647 isotype control, used to establish the cut-off point for CD4+ lymphocytes expressing FOXP3 intracellularly; (**E**). dot plot logarithmic scale (CD4 V450)/FOXP3 Alexa Fluor 647 for CD4+ lymphocytes with intracellular expression of the FOXP3 antigen; (**F**). dot plot (dot plot) log scale CD25 PE-Cy7/CD134 FITC defining CD134-expressing Tregs among CD4+ T cells.

Taking into account the parameters of cell size (forward scatter, FSC) and granules inside the cell (side scatter, SSC), gates lymphocytes and monocytes were created. The gate for regulatory T cells (Tregs) was set based on the CD4+CD25+FoxP3+ immunophenotype and the percentage of cells expressing the CD134 antigens was analyzed (Figure 1).

### 2.3. Statistical Analysis

Statistical analysis was performed using MedCalc (version 15.8 PL) and Statistica (version 13 PL). The normality of the distribution of continuous variables was tested using the D’Agostino–Pearson test. The distribution of continuous variables between the study groups was compared using the Mann–Whitney U test. Comparisons of categorized variables between the evaluated groups were performed based on the chi-square test with Yates’ correction (more than two compared categories of a variable/group) or the Fisher exact test (for comparisons of two categories of a variable in two groups). Results for which *p* was less than 0.05 were interpreted as statistically significant.

## 3. Results

Patients qualified for the final analysis were divided into two subgroups:

1/. The first group consisted of patients with a mild form of isolated hypertension manifesting after the 20th week of pregnancy—30 pregnant women;

2/. The second group included 30 pregnant women with pre-eclampsia.

The control (*n* = 48) and study group (*n* = 60) did not differ statistically significantly in terms of basic demographic and clinical variables, such as maternal age, number of pregnancies, number of births or number of miscarriages. These differences were also not found among women with different forms of hypertension. The median was used as a measure of data clustering, while data dispersion was represented by the interquartile range and/or minimum–maximum range.

These characteristics and a comparison of the study and control groups in terms of basic demographic and clinical variables are presented in Table 2.

## 4. Discussion

Diseases and factors predisposing to gestational hypertension include insulin resistance, diabetes mellitus, obesity, thrombophilia, chronic hypertension, kidney disease, gestational trophoblastic disease, hyperhomocysteinemia, protein C or S deficiency, increased testosterone levels and autoimmune disorders (collagenosis, antiphospholipid syndrome) [2,37,38]. It is also known that both extremely low and advanced age at the time of pregnancy significantly increase the risk of developing hypertension [39,40]. Other recognized risk factors are multiple pregnancy, fetal hydrops and the presence of chromosomal aberrations such as triploidy or trisomy 13 [9,41,42]. There was also a correlation between the higher incidence of pre-eclampsia and the male sex of the fetus in relation to pregnancies with female fetuses [41]. Among the genetic factors predisposing to pre-eclampsia, the participation of many genes and their polymorphisms is suggested [1,3].

Impaired remodeling of the spiral arteries is the cornerstone of pathophysiology of hypertensive disorders in pregnancy [26,27]. Disturbed blood flow through the uteroplacental unit results in early placental hypoxia. As a consequence of ischemia and hypoxia of placental tissues, damage to endothelial cells [43] and the release of a number of mediators into the maternal circulation is observed. These factors stimulate the maternal immune system and ultimately lead to systemic inflammation [44]. Particularly large amounts of TNF-α and IL-1 are released from trophoblast cells and macrophages. These cytokines are responsible for increasing vascular permeability, the expression of adhesion molecules and the level of plasminogen activator inhibitor and activated platelets [42,45]. Inflammation and immune cells play an important role in the process of embryo implantation, placental development and birth. Imbalance between Th1, Th2, Treg and Th17 cells locally at the decidual side and in peripheral blood are associated with pregnancy complications such as unexplained recurrent miscarriage, pre-eclampsia, intrauterine growth restriction or preterm delivery [28].

During physiological pregnancy, due to the increase in mitochondrial activity in the placental cells, there is an excessive production of reactive oxygen species (ROS) causing oxidative stress. It has been observed that in pregnancy complicated by hypertension, this process is intensified [9,46,47]. Inflammatory cells such as macrophages and granulocytes may release ROS that can aggravate oxidative stress [48]. Low levels and activity of tissue antioxidants such as antioxidant vitamins (A, C and E) and antioxidant enzymes (glutathione peroxidase (GPx), superoxide dismutase (SOD), catalase, reductase) were confirmed in pregnant women with pre-eclampsia [9,46,47]. ROS can affect the polarization of T cells and cytokine secretion [48].

The presence of chemokine receptors such as CCR5, CCR6 or CCR7 on Tregs let them move to sites of inflammation and participate in the suppression of the immune response [49]. Studies in animal models have shown that in Tregs deficiency, permanent impairment of spiral artery remodeling is observed, leading to reduced placental perfusion and FGR [26,27,50]. Reducing the Treg population in early pregnancy later leads to abnormal uterine artery function associated with increased production of endothelin 1, which is an important vasoconstrictor [27]. Tregs have the ability to reduce inflammation and oxidative stress in blood vessels, endothelial dysfunction, infiltration of aortic macrophages and T cells to lower blood pressure and prevent tissue damage that occurs with hypertension [51,52]. Therefore, it can be concluded that Tregs have a strong antihypertensive effect with regard to their ability to produce anti-inflammatory cytokines such as IL-10. The symptoms of pre-eclampsia are believed to be T-cell dependent, i.e., athymic nude animals that do not produce T cells cannot be induced to show symptoms of pre-eclampsia. Symptoms occur only after the administration of Th17 cells and are alleviated after the administration of Tregs obtained from healthy pregnant individuals. [29,53]. Rat models of preeclampsia indicate that Tregs prevent the progression of symptoms observed in the course of preeclampsia.

In women with preeclampsia, the population of decidual and circulating Tregs is reduced [21,36,54] and their immunosuppressive function is impaired [55,56]. Among our patients, the population of CD4+CD25+Foxp3+ cells was slightly lower in the group of women with hypertension. Within the study group, however, a significantly higher percentage of these cells was found in women with pre-eclampsia than in patients without proteinuria. It is difficult to refer to the above result, as no one has made a similar analysis so far. Perhaps the severity of systemic disorders associated with hypertension in pregnancy does not depend on the size of the Tregs population itself, but on their disturbed function. Tregs recruitment and their programming may be affected by many factors, from cytokines, hormones, microRNAs to the microbiome of the reproductive tract [57,58,59]. In a pro-inflammatory environment, pTregs are plastic and may be unstable, thus acquiring phenotypic and functional features consisting in the expression of pro-inflammatory cytokines, typical of effector T cells [60,61].

The specific functions of T cells depend on signals that reach the cells through receptors and costimulatory molecules [62]. We have shown that in patients with hypertension and proteinuria, the percentage of Tregs with CD123 (OX40) expression is significantly higher than in patients with isolated hypertension. OX40 inhibits the differentiation of Treg lymphocytes, negatively affects Foxp3 expression, and OX40 costimulation abolishes the suppressor functions of natural Foxp3 Tregs and prevents the transformation of effector T cells into new FoxP3+ Tregs [35,63]. The loss of suppressor function induced by OX40 stimulation is not due to impaired proliferation of Foxp3+ Tregs or death of these cells, but is related to the blocking of Foxp3 gene expression.

The expression of the OX40 receptor on the surface of activated T cells is transient, and the OX40-OX40L interaction is necessary for optimal effector functions [64,65]. Costimulatory signals from OX40 transmitted to conventional T cells promote division and long-term survival of CD4 T cells, and increase effector cell expansion and memory cell development [32,33,66,67]. In addition, it has been shown that the increase in OX40/OX40L expression promotes the production of cytokines by effector cells [68].

OX40 is a pro-inflammatory receptor; therefore, OX40/OX40L interactions seem to play an important role in the development of many inflammatory and autoimmune diseases. On one hand, deliberate stimulation of OX40 in vivo may impair tolerance to protein antigens [69]. On the other hand, blocking OX40 co-stimulation reduces cytotoxicity and allows allograft survival [70]. Similarly, in autoimmune diseases the blockade of the OX40/OX40L system resulted in the alleviation of symptoms accompanying the disease [71]. So far, it has been confirmed, among others, that blocking the OX/OX40R interaction prevents the differentiation of CD4+ T cells and the development of inflammation in models of multiple sclerosis, autoimmune diseases of the gastrointestinal tract, GvHD, rheumatoid arthritis and atopic dermatitis [72,73,74,75,76]. These data suggest that OX40 probably exerts a major influence on the nature of the immune response.

Current studies and clinical trials using humanized antibodies against OX40 have confirmed the safety and increased antitumor response in many types of cancer [77].

The polymorphism of the OX40L gene is associated with susceptibility to atherosclerosis in humans [78]. The pathological effect of the OX40L-dependent effect on the functions of the vascular endothelium in the systemic arteries has also been demonstrated [79].

However, so far, little research has been devoted to the role of the OX40 molecule in pregnancy and pregnancy-induced hypertension. Increased plasma concentrations of OX40L are observed during pregnancy compared to the nonpregnant women [80]. The authors noted that the concentration of the OX40 ligand behaves similarly to the population of Tregs in pregnancy. It increases significantly at the beginning, peaks in the second trimester and decreases before delivery. There were no significant differences in the expression of OX40 and OX40L mRNA in peripheral blood between women with a history of recurrent miscarriages and those without a bad obstetric history, and only plasma OX40 concentrations were significantly higher in women with miscarriages [81].

A paper published in 2022 presents the results of the study, which showed an increased level of expression of OX40 and CXCR5 on decidual immune cells in the course of pre-eclampsia compared to physiological pregnancy [82]. The authors of the publication emphasized the role of OX40 and CXCR5 overexpression in the production of autoantibodies by B lymphocytes, which is one of the components of the pathogenesis of preeclampsia.

The real relationship between the excessive expression of OX40 on CD4+CD25+FoxP3+ cells and the severity of changes in the maternal body in the course of pregnancy-induced hypertension certainly requires further research. Similarly, the issue of the possible use of agents blocking the OX40/OX40L binding, which could limit the development of dangerous symptoms of pre-eclampsia, needs more studies.

In conclusion, our data clearly demonstrate that OX40 expression in Tregs seems to be related to a more serious type of hypertensive disorder in pregnant women, and these new findings may have important therapeutic implications in the clinic.

## Figures and Tables

**Figure 2:**
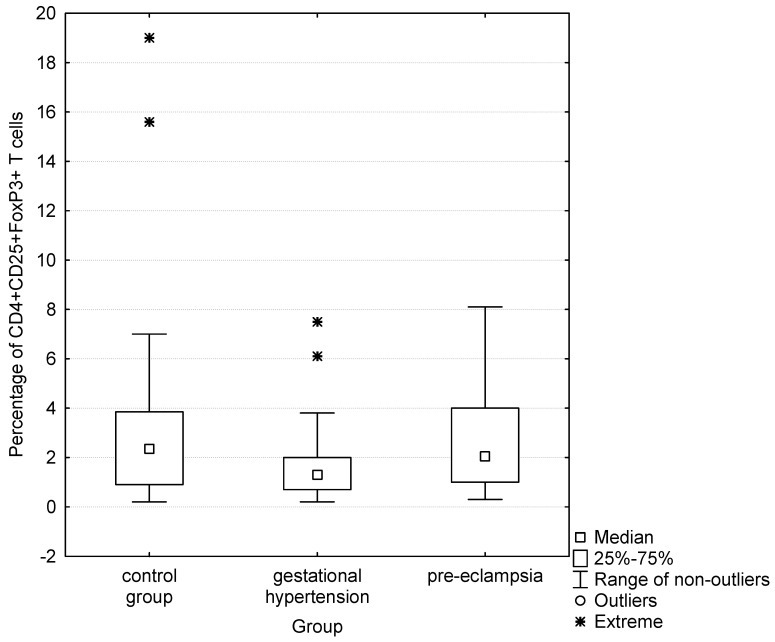
Comparison of percentages of CD4+CD25+FoxP3+ cells in control group, gestational hypertension and pre-eclampsia.

**Figure 3:**
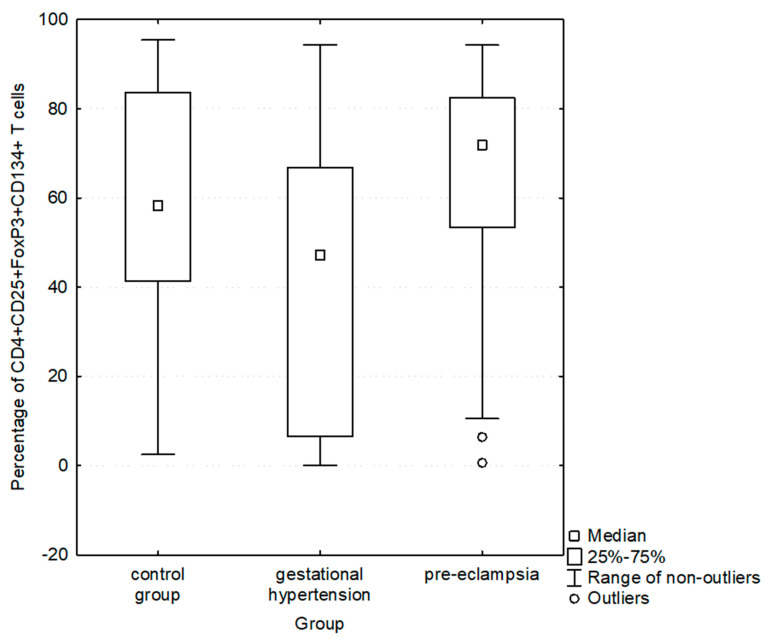
Comparison of percentages of CD4+CD25+FoxP3+ cells with CD134 antigen expression in control group, gestational hypertension and pre-eclampsia.

**Table 1:** Division of women into groups depending on inclusion and exclusion criteria.

	Inclusion Criteria	Exclusion Criteria
**Study group n = 60**	Gestational hypertension	Chronic hypertension Superimposed pre-eclampsia Other chronic maternal diseases Threatening preterm delivery PROM Signes of infection
	Pre-eclampsia	
**Control group n = 48**	Healthy pregnancy	Any maternal disease or pregnancy complication

**Table 2:** Characteristics and comparison of the study and control groups in terms of basic demographic and clinical variables.

Variable	Control Group (n = 48) n (%) or Median [Interquartile Range] (Min-Max)	Study Group (n = 60) n (%) or Median [Interquartile Range] (Min-Max)	*p* or *Chi*^2^*, p*	Gestational Hypertension (n = 30) n (%) lub Median [Interquartile Range] (Min-Max)	Pre-Eclampsia (n = 30) n (%) lub Median [Interquartile Range] (Min-Max)	*p* or *Chi*^2^*, p*
Maternal age (years)	29 [27–32] (18–40)	31 [27.5–35] (19–42)	0.1425	31 [28–34] (19–41)	30.5 [26–36] (21–42)	0.9410
Maternal age and parity Primiparas and multiparas 21–34 yo Primiparas ≤20 yo Multiparas ≥35 yo	43 (89.6%) 5 (10.4%)	50 (83.3%) 10 (16.7%)	0.5136	25 (83.3%) 5 (16.7%)	26 (86.7%) 4 (13.3%)	1.0000
Number of pregnancies	2 [1–2] (1–4)	1 [1–2] (1–3)	0.1492	1 [1–2] (1–3)	1 [1–2] (1–3)	0.3942
Number of pregnancies 1 2 3 4	23 (47.9%) 21 (43.7%) 3 (6.2%) 1 (2.1%)	38 (63.3%) 17 (28.3%) 5 (8.3%) -	0.2280	17 (56.7%) 11 (36.7%) 2 (6.7%)	22 (70%) 6 (20%) 2 (10%)	0.3514
Parity	1 [1–2] (1–3)	1 [1–2] (1–3)	0.3087	1 [1–2] (1–2)	1 [1–2] (1–3)	0.7182
Parity 1 2 3	29 (60.4%) 17 (35.4%) 2 (4.2%)	42 (70%) 16 (26.7%) 2 (3.3%)	0.5796	20 (66.7%) 10 (33.3%) -	22 (73.3%) 6 (20%) 2 (6.7%)	0.2128
Number of miscarriages	0 [0–0] (0–1)	0 [0–0] (0–2)	0.2253	0 [0–0] (0–2)	0 [0–0] (0–1)	0.4096
History of miscarriage 0 1 2	39 (81.2%) 9 (18.8%) -	53 (89.9%) 5 (8.5%) 1 (1.7%)	0.2043	26 (86.7%) 3 (10%) 1 (3.3%)	26 (93.1%) 2 (6.9%) -	0.5482
Gestational age at sampling	40 [39–40] (37–41)	38 [36–39] (24–41)	<0.0001	39 [38–40] (33–40)	36.5 [32–38] (24–41)	0.0001
Gestational age at delivery	40 [39–41] (37–41)	38 [37–39] (28–41)	<0.0001	39 [38–40] (33–41)	38 [34–39] (28–41)	0.0136

In the group of women with preeclampsia compared to the group with gestational hypertension, significantly higher percentages of CD4+CD25+FoxP3+ cells (medians were: 2.1 vs. 1.4, respectively; *p* = 0.0389; Table 3, Figure 2) and percentages of CD4+CD25+FoxP3+ cells expressing the CD134 antigen (medians were: 71.9 vs. 43.2, respectively; *p* = 0.0014; Table 3, Figure 3) were observed.

**Table 3:** Characteristics and comparison of immune parameters of peripheral blood depending on the form of hypertension in the course of pregnancy.

Variable	Control Group (n = 48) Median [Interquartile Range] (Min-Max)	Study Group (n = 60) Median [Interquartile Range] (Min-Max)	*p*	Gestational Hypertension (n = 30) Median [Interquartile Range] (Min-Max)	Pre-Eclampsia (n = 30) Median [Interquartile Range] (Min-Max)	*p*
Peripheral lymphocytes (percentage)	45.3 [37–60.9] (14.6–72.9)	47.5 [39.4–54.7] (16.6–79.1)	0.9501	47.6 [41.6–54.7] (23.4–61.7)	47.3 [37.3–54] (16.6–79.1)	0.7159
Percentage of lymphocytes CD4+FoxP3+	37.4 [31.8–42.2] (17–56)	37.9 [31.9–42.4] (18.2–60)	0.7666	38.2 [36.1–42.3] (21.4–53.4)	37.3 [27.8–42.8] (18.2–60)	0.3912
Percentage of CD4+CD25+FoxP3+	2.4 [0.9–3.9] (0.2–19)	1.9 [0.8–3.4] (0.2–8.1)	0.1677	1.4 [0.7–2] 90.2–7.5)	2.1 [1–4] (0.3–8.1)	0.0389
Percentage of CD4+CD25+FoxP3+ expressing CD134+	58.3 [41.3–83.7] (2.5–95.4)	59.5 [13.3–75.8] (0–94.3)	0.3180	43.2 [6.5–65.6] (0–94.3)	71.9 [53.4–82.4] (0.6–94.3)	0.0014

Groups of women with gestational hypertension (*n* = 30) and pre-eclampsia (*n* = 30) did not differ statistically significantly in terms of basic demographic and clinical variables such as maternal age (medians were: 31 years and 30.5 years, respectively) number of pregnancies (both medians were 1), number of births (both medians were 1), number of miscarriages (both medians were 0).

## Data Availability

The data presented in this study are available on request from the corresponding author.
